# 

**Published:** 1860-04-01

**Authors:** 


*In a few days will be published, One Volume 8vo,*

### A TREATISE

ON

### OBSCURE

### DISEASES OF THE BRAIN

AND

### MIND:

THEIR

INCIPIENT SYMPTOMS, PATHOLOGY, DIAGNOSIS,
TREATMENT, AND PROPHYLAXIS

BY FORBES WINSLOW, M.D.
HON. D.C.L. OXON.

---

ORDERS FOR THIS WORK ARE TO BE TRANSMITTED TO

JOHN CHURCHILL, PUBLISHER, NEW BURLINGTON STREET,
LONDON.

